# Cooperative Spectrum Sensing Schemes with the Interference Constraint in Cognitive Radio Networks

**DOI:** 10.3390/s140508037

**Published:** 2014-05-05

**Authors:** Tri-Nhu Do, Beongku An

**Affiliations:** 1 Department of Electronic & Computer Engineering in Graduate School, Hongik University, Sejong 339-701, Korea; E-Mail: dotrinhu@gmail.com; 2 Department of Computer & Information Communications Engineering, Hongik University, Sejong 339-701, Korea

**Keywords:** cognitive radio, spectrum sensing, underlay, amplify-and-forward, decode-and-forward

## Abstract

In this paper, we propose cooperative spectrum sensing schemes, called decode-and-forward cooperative spectrum sensing (DF-CSS) scheme and amplify-and-forward cooperative spectrum sensing (AF-CSS) scheme, in cognitive radio networks. The main goals and features of the proposed cooperative spectrum sensing schemes are as follows: first, we solve the problem of high demand for bandwidth in a soft decision scheme using in our proposed schemes. Furthermore, the impact of transmission power of relaying users which is determined by the interference constraint on sensing performance of cooperative spectrum sensing schemes is also investigated. Second, we analyze the sensing performance of our proposed cooperative spectrum sensing schemes in terms of detection probability and interference probability, respectively. We take into account the interference caused by secondary user (SU) to primary user (PU) in the case that the transmission power of the relaying users exceeds a predefined interference constraint assigned by the primary user. The simulation results show that in cooperative spectrum sensing schemes the total sensing performance depends not only on the interference tolerance level, but also on the relay protocols used. We also prove that high transmission power of relaying users increases the interference between the secondary networks and the primary network.

## 1. Introduction

The demand for radio spectrum has rapidly increased since the number of wireless applications and standards are increasing significantly. However, the current static spectrum allocation policy has caused a shortage of spectrum resources because almost all the spectrum has been allocated and there is no new spectrum for new wireless services. On the other hand, actual measurements by the Federal Communications Commission (FCC) have shown that the allocated spectrum in the US is largely under-utilized [1]. In order to improve the utilization of the finite spectrum sources, a new intelligent communications system named cognitive radio (CR) is proposed. Cognitive radio (CR) technology enables unlicensed wireless devices to use the under-utilized spectrum by obtaining the necessary observations about their surrounding radio environment [2]. Therefore, the CR technique allows a more efficient utilization of the spectrum by operating in the idle channel.

In CR, the secondary (unlicensed) users opportunistically or concurrently access the spectrum allocated to primary (licensed) users. A prerequisite to this secondary access is that does not cause interference to the primary system. This requirement makes spectrum sensing a key function in cognitive radio systems. Among common spectrum sensing techniques, energy detection has been widely applied because of its simplicity and efficiency. However, energy detectors are susceptible to noise uncertainty in low SNR regimes and reliable detection of the primary user is not possible, even with a large amount of sensing time [3]. On the other hand, a critical issue in cognitive radio is to reliably and quickly detect the presence of the primary users. In addition, the performance of spectrum sensing is limited by multipath fading and shadowing which are the fundamental characteristics of wireless channels. To overcome these challenges, cooperation among SUs to perform spectrum sensing has been proposed in the literature.

In cooperative spectrum sensing, information from different CR users is combined to make a decision on the presence or absence of the primary user. Cooperation among secondary users is usually coordinated by a fusion center (FC). Fusion schemes can be broadly categorized into soft decision schemes and hard decision schemes, depending on the type of sensing information being sent by the SUs [4,5]. In this paper, we focus on soft decision schemes. In [6], a soft decision scheme with relay using an amplify-and-forward protocol has been proposed. In that scheme, the secondary user with higher sensing reliability acts as a relay to help the other users that wants to use the primary spectrum band. By exploiting all the information available in both sensing and reporting phases in similar schemes in [6], the authors in [7] obtained a better sensing performance. Generally, soft decision combination has better sensing performance, but requires a much larger bandwidth for transmission of the sensing information [4]. However, the question about how to reduce the demand of bandwidth for reporting channel in soft decision scheme was not considered in [6,7].

In this paper, we develop an efficient cooperative sensing scheme based on the concept of the underlay approach in cognitive radio systems. The underlay approach to cognitive radio allows the secondary user to use the spectrum of the primary user simultaneously under the constraint that the interference caused to the primary user by secondary user does not degrade its communication [8]. More particularly, in [9], the outage probability of the underlay cognitive radio with best relay selection was investigated. A similar work in which the authors evaluated the interference probability of secondary transmissions on a primary receiver was reported in [10]. However, these works just focus on the data transmission, but not on application to spectrum sensing in cognitive radio networks. In addition, underlay cognitive radio still has a weak point in that cognitive users are limited to short range communications due to the transmission power constraints [8]. Hence, for the application with long distance transmission and high data rate, the secondary users should freely use the licensed spectrum with a normal transmit power level as the primary user to satisfy the QoS requirements.

Besides, in centralized cooperative sensing, the time delay for reporting sensing data will be large when the network size is expanded. We also consider this issue in our proposed cooperative spectrum sensing schemes by using best relay selection (BRS) protocol [11]. Among the various cooperative strategies, transmission based on relay selection has been shown to provide substantial cooperative gains as well as being spectrally and costly more efficient than repetitive transmission techniques [9]. Therefore, a best relay selection (BRS) has been commonly applied in several CR networks subject to a spectrum sharing condition, either assuming decode-and-forward (DF) relays or amplify-and-forward (AF) relays. It has been shown that exploiting user cooperation significantly enhances the cognitive system performance [9,10]. In this paper, we study the effect of the decode-and-forward, amplify-and-forward and best relay selection protocols on the spectrum sensing capabilities of cognitive radio network.

The main difference and typical characteristic of our proposed schemes with other cooperative schemes, *i.e.*, the schemes presented in [6,7], are as follows: in this paper, we propose CSS schemes that rely on the concept of soft sensing and underlay cognitive radio, in which the underlay approach is adopted as a method for cooperative users to exchange their sensing data. Most cooperative spectrum schemes assume that there is a dedicated reporting channel for SUs to exchange their sensing data, but this assumption makes the soft combination schemes consume more radio resources. By using an underlay approach, our proposed schemes do not need the dedicated channel that is used for SUs to exchange their collected data. To the best of the authors' knowledge, there are no previous works that use an underlay approach as a method for exchanging data between cooperative SUs in spectrum sensing.

The organization of the paper is as follows: in Section 2, we describe in detail the basic concepts of the proposed cooperative spectrum sensing schemes and the system model. In Section 3, we then study and present theoretical analysis of the sensing performance of our proposed schemes and the probability of interference between the secondary and primary networks. In Section 4, we show the effect of interference constraints on the performance of cooperative detection schemes by using both theoretical and simulation results. Finally, in Section 5 we present our conclusions.

## 2. The Basic Concept of the Proposed Schemes and System Model

In this section, we describe in detail the basic concepts of the proposed cooperative spectrum sensing schemes. In [4], it was shown that soft decision fusion schemes have the ability to achieve better performance compared to hard decision schemes. However, under the same channel conditions, the bandwidth cost of sending one bit per decision in a hard decision scheme is smaller than that of sending the collected data of a primary signal for a soft decision scheme. Moreover, since this scheme needs more extra bandwidth, the bandwidth demand becomes high when the number of SUs in the network is huge. Another idea for the reporting channel between cooperative secondary users was proposed in [12]. The transmission of the sensing information can be done using an unlicensed band such as the industrial, scieic and medical (ISM) radio bands. However, the SUs may suffer severe interference from a variety of devices using the ISM band. To overcome this weak point of soft decision schemes, we propose the cooperative spectrum sensing schemes using soft decision, in which the collected data are exchanged between the cooperative secondary users by using an underlay approach in a cognitive radio system. By using the underlay approach, the proposed schemes do not need a dedicated reporting channel for exchanging sensing data between cooperative secondary users.

In the underlay approach, the secondary user is allowed to use the licensed spectrum simultaneously with the primary user only when the interference that is caused by the secondary user is less than the interference level which the primary user can tolerate. Let *I_t_* be the interference threshold of the primary user. Hence, the transmission power of the secondary user is constrained not to exceed this interference threshold *I_t_*. This approach may not only reduce the complexity of reporting channel management, but also solve the demand for bandwidth in soft decision schemes. However, since the underlay cognitive radio network operates in a fixed time-division multiple-access (TDMA) mode, the cooperative spectrum sensing requires a larger sensing time that involves sending data to the fusion center when the number of collaborating users is higher. In order to satisfy the essential requirements for the fast spectrum sensing and reliable signal detection, the best relay selection scheme is adopted in our proposed schemes as the method to solve this issue. Besides, in order to improve the sensing performance, we use AF and DF protocol to achieve the cooperative diversity.

**Figure 1.**
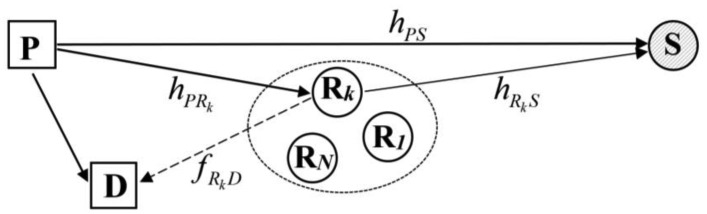
Cognitive radio system model with the coexistence of a secondary network under a primary network.

Let's consider a cognitive radio system with the coexistence of primary and secondary networks as depicted in Figure 1. The primary network contains a primary transmitter P and a primary receiver D. The hypotheses of the absence and the presence of the primary user P are denoted as *H_0_* and *H_1_*, respectively. We assume that the primary status is not changed during the sensing period. The secondary network is represented by one secondary user S and *N* secondary relays, denoted by ***R*** = {R*_k_* | *k* = 1,2,…,*N*}. Herein, S wants to use the primary spectrum. All *N* secondary users R*_k_* are available to help S sense the primary spectrum. In addition, all users in the entire system are equipped with one antenna and operate in half duplex mode.

We assume that each link between any two users is modeled as a Rayleigh fading channel and pairwise independent. We also assume that each user has access to its instantaneous channel state information (CSI). In addition, we assume that all the SUs have the knowledge of the average channel gain of the link from itself to other users in both primary and secondary networks. We assume that the secondary relays R*_k_*'s have an identical average SNR of the received primary signal. This is facilitated by allowing pilot symbols to be transmitted at regular intervals. The identical SNR assumption can be practical when all the relays are gathered in a small area.

In the spectrum sensing process, the sensing duration in each fixed time slot is divided into two sub-phases. In the first sub-phase, each SU collects the PU's signal. In the second sub-phase, by using the best relay selection scheme, only the relay which has the highest instantaneous signal-to-noise ratio (SNR) of the signal that received from primary user sends its collected data to S by using either a decode-and-forward protocol or an amplify-and-forward protocol. At the end of sensing process, S combines the received signals from the primary user and best relaying user using the maximum ratio combining (MRC) method, and makes the final decision about the status of the primary user by using an energy detector. Throughout the paper, we assume that the secondary user S has strong computation ability so that it can make the local decision by using its own sensing data and the relay data. Here, the secondary user S also plays a role as the fusion center.

Under underlay approach, the transmission power of the best relay R*_k_* is calculated based on the interference constraint *I_t_* that is defined by the primary receiver D and the instantaneous CSI of the interference link between the secondary relay R*_k_* and the primary receiver D. In practice, it is difficult to estimate perfectly the channel state information (CSI) between secondary and primary networks (e.g., due to mobility between SUs and PUs). We take into account this scenario in terms of interference probability that is denoted by *P_I_* of the secondary relay transmissions on the primary receiver D. In particularly, we analyze the interference caused by the secondary user to the primary user since the secondary transmission power exceeds the predefined interference threshold *I_t_*.

By using the underlay approach and best relay selection, the main objective we focus on in this paper is how to reduce radio resources such as bandwidth, and sensing time for a soft combination scheme with a large number of SUs in the network. With that purpose, while we adopt a simple cooperative scheme and fusion rule in our scheme to facilitate the analysis work, we do not focus on the improvement of sensing performance to compare with related works. For the whole paper, *E*[.] denotes the statistical expectation operator and Pr(A) is the probability of an arbitrary event A.

## 3. The Theoretical Analysis

### 3.1. Non-Cooperation Spectrum Sensing Scheme

In the non-cooperative spectrum sensing scheme, each secondary user decides independently the status of the primary user. Let us assume that the primary user P transmits signal *x_P_* (*E*[|*x_P_*|^2^] = 1) to the primary receiver D with a fixed power *P_P-Tx_* in a certain time slot *t*. In the meantime, all the secondary users in the secondary network also listen to the primary signal from P. The signals received by SU*_i_*, *i* ∈ {*S*, *R*_1_,*R*_2_,…*R_N_*} from primary user P can be expressed as:

(1) $$y_{i}=\theta_{i}\sqrt{P_{P-\text{Tx}}}h_{Pi}x_{P}+n_{i}$$

where the time index *t* is dropped for notational convenience, *h_Pi_* which is modeled as a complex Gaussian random variable with zero mean denotes the instantaneous channel gain of the channel from the primary user P to a secondary user SU*_i_*, and *n_i_* denotes the additive Gaussian noise with zero-mean and unit variance. Also, we assume that all the instantaneous channel gain *h_Pi_'s* and the additive Gaussian noise *n_i_'s* in the secondary network are independent. Besides, *θ_i_* denotes the primary user indicator such that *θ_i_* = *1* implies the primary user is present and *θ_i_* = *0* implies the primary user is absent.

The detection of primary user is a binary hypothesis testing problem, which can be written as follows:

$$\begin{array}{cc} H_{0}:\theta_{i}=0, & \text{the primary user is absent},\\ H_{1}:\theta_{i}=1, & \text{the primary user is present}. \end{array}$$

In this paper, we utilize the energy detector (ED) [3] as a method of local spectrum sensing. The ED forms the statistics:

$$Y_{i}={\left|y_{i}\right|}^{2}$$

and compares with the decision threshold *λ_i_* which is determined by a pre-specified probability of false alarm *P_f,i_* = *β*. If the observation statistic is greater than a decision threshold *λ_i_*, namely *Y_i_* > *λ_i_*, then the secondary user SU*_i_* decides that the primary user is present, otherwise the secondary user SU*_i_* decides that the primary user is absent. The expected signal power *γ_i_* of *Y_i_* (*i.e.*, *γ_i_* = E[*Y_i_*]) is calculated as:

(3) $$\gamma_{i}=\theta_{i}P_{i-Rx}+1$$

where *P_i-Rx_* = E[*P_P-Tx_*|*h_Pi_*|*^2^*] refers to the received signal power at SU*_i_* from the primary user P.

Since *y_i_* is complex Gaussian random variable, *Y_i_* follows exponential distribution with parameter *1*/*γ_i_*. The detection probability *P_d_*_,_*_i_* of the secondary user SU*_i_* under the non-cooperative spectrum sensing protocol is given by:

(4) $$\begin{array}{ll} P_{d,i} & =\mathrm{Pr}\left(Y_{i}>\lambda_{i}|H_{1}\right)\\ & =\int_{\lambda_{i}}^{\infty }\frac{1}{\gamma_{i|H_{1}}}\exp\left(-\frac{t}{\gamma_{i|H_{1}}}\right)dt\\ & =\exp\left(-\frac{\lambda_{i}}{P_{i-Rx}+1}\right) \end{array}$$

where *λ_i_* is obtained from false alarm probability *P_f,i_* as follows:

(5) $$\begin{array}{ll} P_{f,i} & =\mathrm{Pr}\left(Y_{i}>\lambda_{i}|H_{0}\right)\\ & =\int_{\lambda_{i}}^{\infty }\frac{1}{\gamma_{i|H_{0}}}\exp\left(-\frac{t}{\gamma_{i|H_{0}}}\right)dt\\ & =\exp\left(-\lambda_{i}\right) \end{array}$$

Where *γ*_*iH*_1__ and *γ*_*iH*_10__ are the expected signal power *γ_i_* of *Y_i_* under hypothesis *H_1_* and *H_0_*, respectively.

Therefore, under a constant probability of false alarm *P_f,i_* = *β*, the detection threshold *λ_i_* of secondary users SU*_i_*, according to Equation (5), is given by:

$$\lambda_{i}=\ln\frac{1}{\beta }$$

### 3.2. Decode-and-Forward Protocol Based Cooperative Spectrum Sensing (DF-CSS) Scheme

In this subsection, we mathematically describe the cooperative spectrum sensing scheme using a decode-and-forward protocol in a cognitive radio system. Then, we derive the detection probability *P_d,S,DF_* and the false alarm probability *P_f,S,DF_* of the secondary user S.

The sensing process is conducted in two sub-phases. In the first sub-phase, all the secondary users listen to the signal from the primary user P. The received signal at each secondary user from the primary user is described as in Equation (1). Then, all the secondary relays will decode their received signals. In the second sub-phase, without loss of generality, consider that only a candidate relay R*_k_*, *k*^∈^{1,2,…, *N*}, is selected to forward its decoded result to the secondary user S. Herein, we utilize the instantaneous channel gain of the link from the primary user P to secondary rely R*_k_* to determine which “best” relay will be selected to send its own data to secondary user S in each sensing period. As a consequence, the best relay selection criterion can be written as:

(7) $$\begin{array}{ll} \text{Best relay} & =\arg\underset{R_{k}\in R}{\max}SNR_{R_{k}}\\ & =\arg\underset{R_{k}\in R}{\max}\frac{P_{R_{k}-Rx}{\left|h_{PR_{k}}\right|}^{2}}{\sigma_{i}^{2}}\\ & =\arg\underset{R_{k}\in R}{\max}{\left|h_{PR_{k}}\right|}^{2} \end{array}$$

where *h_PRk_* which is modeled as a complex Gaussian random variable with zero mean denotes the instantaneous channel gain of the channel from the primary user P to a secondary relay R*_k_*, and *σ_i_^2^* is the noise variance which is assumed to be unit.

With the aforementioned assumption that all the relays are gathered in a small area, the average received signal power *P_Rk_*_−_*_Rx_* at the relays R*_k_*'s can be assumed identical. We can see that there are two possible scenarios for this scheme. In the first scenario, if primary user P is absent, then R*_k_* will keep silent because there is no primary signal to decode. Therefore, S cannot get any help from R*_k_* and make a decision by itself. On the other hand, in the second scenario, if primary user exists, R*_k_* will decode the received signal and sends it to S. We assume that R*_k_* can always fully decode the primary signal if the primary user is present. In the underlay cognitive radio structure, the transmission power at R*_k_* may be expressed as:

(8) $$P_{R_{k}-\text{Tx}}=\frac{I_{t}}{{\left|f_{R_{k}D}\right|}^{2}}$$

where *f_R_k_D_* which is modeled as a complex Gaussian random variable with zero mean denotes the instantaneous channel gain of the interference channel from the secondary relay R*_k_* to the primary receiver D.

Notice that the instantaneous channel gains *h_ij_* and *f_ij_*, are assumed to be zero-mean complex Gaussian random variables, respectively, where *i*^∈^{P, S, R*_k_*}, *j*^∈^{R*_k_*, D}. Therefore, the instantaneous channel gains |*h_R_k_S_*|^2^ and |*f_R_k_D_*|^2^ of these links follows exponential distribution with the parameters:

(9) $$\begin{array}{cc} \frac{1}{\psi_{R_{k}S}}=\frac{1}{E\left[{\left|h_{R_{k}S}\right|}^{2}\right]}, & \frac{1}{\psi_{R_{k}D}}=\frac{1}{E\left[{\left|f_{R_{k}D}\right|}^{2}\right]} \end{array}$$

respectively.

Finally, the secondary user S combines two signals, one is from the primary user P and the other is from the best secondary relay R*_k_* using the maximum ratio combining (MRC) method, and does the spectrum sensing by using energy detector as we mentioned in Section 3.1. The received signal at S can be written as:

(10) $$y_{S,DF}=\theta_{S}\sqrt{P_{P-\text{Tx}}}h_{PS}x_{P}+\theta_{R_{k}}\sqrt{P_{R_{k}-\text{Tx}}}h_{R_{k}S}x_{P}+n_{S}$$

where *h_Rk_*_S_ which is modeled as a complex Gaussian random variable with zero mean denotes the instantaneous channel gain of the channel from the secondary relay R*_k_* to a secondary user S, and *θ_S_* denotes the primary user indicator of the secondary user S, and *θ_Rk_* denotes the primary user indicator of all the secondary relays R*_k_*.

The energy detector forms the statistics *Y_S,DF_* = |*y_S,DF_*|^2^ and compares it with its threshold *λ_S,DF_* which is determined by a pre-assigned false alarm probability *β*. In addition, the expected signal power *γ_S,DF_* of *Y_S,DF_* is calculated as:

(11) $$\gamma_{S,DF}=E\left[Y_{S,DF}\right]=\theta_{R_{k}}I_{t}\frac{{\left|h_{R_{k}S}\right|}^{2}}{{\left|f_{R_{k}D}\right|}^{2}}+\theta_{S}P_{S-Rx}+1$$

where *P*_*S*−*Rx*_ refers to the received signal power at secondary user S and secondary relay R*_k_* from the primary user P. Let:

(12) $$z=\frac{{\left|h_{R_{k}S}\right|}^{2}/\psi_{R_{k}S}}{{\left|f_{R_{k}D}\right|}^{2}/\psi_{R_{k}D}}$$

In Equation (12), since the numerator and denominator of *z* are independent and both exponentially distributed with mean one, after some manipulations, probability density function (*pdf*) of *z* is given as:

(13) $$f\left(z\right)=\{\begin{array}{cc} \frac{1}{{\left(1+z\right)}^{2}}, & z>0\\ 0, & z\leq 0 \end{array}$$

Define *ψ* = *ψ_R_k_S_*/*ψ_R_k_D_* then Equation (11) is re-written as:

(14) $$\gamma_{S,DF}=\theta_{R_{k}}I_{t}\psi z+\theta_{S}P_{S-Rx}+1$$

Since *y_S,DF_* given *h_R_k_S_* and *f_R_k_D_* is complex Gaussian random variable, *Y_S,DF_* given *z* follows exponential distribution with parameter *1*/*γ_S,DF_*. By using the theorem of total probability, the detection probability *P_d,S,DF_* of S is given by:

(15) $$P_{d,S,DF}=\mathrm{Pr}\left(Y_{S,DF}>\lambda_{S,DF}|H_{1}\right)\sum_{k=1}^{N}\mathrm{Pr}\left({\left|h_{R_{k}S}\right|}^{2}>\underset{h_{R_{i}S}\in R,i\neq k}{\max}{\left|h_{R_{i}S}\right|}^{2}\right)$$

We first calculate Ω*_k_* as [10]:

(16) $$\begin{array}{ll} \Omega_{k} & =\mathrm{Pr}\left({\left|h_{R_{k}S}\right|}^{2}>\underset{h_{R_{i}S}\in R,i\neq k}{\max}{\left|h_{R_{i}S}\right|}^{2}\right)\\ & =\int_{0}^{\infty }\mathrm{Pr}\left(\underset{h_{R_{i}S}\in R,i\neq k}{\max}{\left|h_{R_{i}S}\right|}^{2}<x\right)f_{{\left|h_{R_{k}S}\right|}^{2}}\;(x)dx\\ & \overset{\left(a\right)}{=}\overset{N-1}{\underset{m=0}{\text{∑}}}\left(\begin{array}{c} N-1\\ m \end{array}\right)\frac{{\left(-1\right)}^{m}}{m+1}\\ & \overset{\left(b\right)}{=}\frac{1}{N} \end{array}$$

In Equation (16), the equality sign (a) is obtained by using cumulative distribution function (CDF) of |*h_R_k_S_*|^2^ and Newton's binomial expansion. The equality sign (b) is derived by using Mathematica software [13]. Hence:

(17) $$\begin{array}{ll} P_{d,S,DF} & =\mathrm{Pr}\left(Y_{S,DF}>\lambda_{S,DF}|H_{1}\right)\overset{N}{\underset{k=1}{\text{∑}}}\Omega_{k}\\ & =\mathrm{Pr}\left(Y_{S,DF}(\theta_{R_{k}}=1)>\lambda_{S,DF}|\theta_{R_{k}}=1,H_{1}\right)\mathrm{Pr}\left(\theta_{R_{k}}=1|H_{1}\right)\\ & +\mathrm{Pr}\left(Y_{S,DF}(\theta_{R_{k}}=0)>\lambda_{S,DF}|\theta_{R_{k}}=0,H_{1}\right)\mathrm{Pr}\left(\theta_{R_{k}}=0|H_{1}\right)\\ & =\beta^{\frac{1}{P_{R_{k}-Rx}+1}}\int_{0}^{\infty }\frac{1}{{\left(1+z\right)}^{2}}\exp\left(\frac{-\lambda_{S,DF}}{I_{t}\psi z+P_{S-Rx}+1}\right)dz\\ & +\left(1-\beta^{\frac{1}{P_{R_{k}-Rx}+1}}\right)\exp\left(\frac{-\lambda_{S,DF}}{P_{S-Rx}+1}\right). \end{array}$$

The derivation of Equation (17) is presented in Appendix 1. The value of threshold *λ_S,DF_* in each sensing period is determined first. We assume that all the secondary users have to maintain the same predefined false alarm probability before doing spectrum sensing, *i.e.*, *P_f,S,DF_* = *P_f_*_,_ *_Rk_* = *β*, then the threshold *λ_S,DF_* is determined as follows:

(18) $$\begin{array}{ll} P_{f,S,DF} & =\mathrm{Pr}\left(Y_{S,DF}>\lambda_{S,DF}|H_{0}\right)\overset{N}{\underset{k=1}{\text{∑}}}\Omega_{k}\\ & =\mathrm{Pr}\left(Y_{S,DF}(\theta_{R_{k}}=1)>\lambda_{S,DF}|\theta_{R_{k}}=1,H_{0}\right)\mathrm{Pr}\left(\theta_{R_{k}}=1|H_{0}\right)\\ & +\mathrm{Pr}\left(Y_{S,DF}(\theta_{R_{k}}=0)>\lambda_{S,DF}|\theta_{R_{k}}=0,H_{0}\right)\mathrm{Pr}\left(\theta_{R_{k}}=0|H_{0}\right)\\ & =\left(1-\beta \right)\exp\left(-\lambda_{S,DF}\right)+\beta \int_{0}^{\infty }\frac{1}{{\left(1+z\right)}^{2}}\exp\left(\frac{-\lambda_{S,DF}}{I_{t}\psi z+1}\right)dz \end{array}$$

The derivation of Equation (18) is presented in Appendix 2.

### 3.3. Amplify-and-Forward Protocol Based Cooperative Spectrum Sensing Scheme

In this subsection, we mathematically describe the cooperative spectrum sensing scheme using an decode-and-forward protocol in a cognitive radio system. Then, we derive the detection probability *P_d,S,AF_* and the false alarm probability *P_f_*_,_*_S_*_,_*_AF_* of the secondary user S.

The sensing process is conducted in two sub-phases time. In the first sub-phase, all the secondary users listen to the signal from the primary user P. The received signal at each secondary user from the primary user is described as in Equation (1). In the second sub-phase, the best relay R*_k_* that is selected according to the best relay selection criterion as in Equation (7) will amplify its received signal and send to the secondary user S without consideration about the status of the primary user. According to the underlay cognitive radio structure and for fair comparison in sensing performance with the DF-CSS scheme, the amplification factor is chosen as:

(19) $$\alpha =\frac{I_{t}}{{\left|f_{R_{k}D}\right|}^{2}}$$

where *f_R_k_D_* which is modeled as a complex Gaussian random variable with zero mean denotes the instantaneous channel gain of the interference channel from the secondary relay R*_k_* to the primary receiver D.

In this scheme, let *θ* denote the primary user indicator for all the secondary user in the network. It is reasonable due to in the first sub-phase, that the secondary S and the relays R*_k_*'s do the cooperation but do not consider the status of the primary user as in a DF-CSS scheme.

Finally, the secondary user S combines two signals, one is from the primary user P, the other is from the best secondary relay R*_k_* using the maximum ratio combining (MRC) method, and does the spectrum sensing by using energy detector. The received signal at S can be written as:

(20) $$\begin{array}{ll} y_{S,AF} & =\theta \sqrt{P_{P-\text{Tx}}}h_{PS}x_{P}+\sqrt{\alpha }h_{R_{k}S}y_{R_{k}}+n_{S}\\ & =\theta \sqrt{P_{P-\text{Tx}}}h_{PS}x_{P}+\sqrt{\alpha }h_{R_{k}S}\left(\theta \sqrt{P_{P-\text{Tx}}}h_{PR_{k}}x_{P}+n_{R_{k}}\right)+n_{S}\\ & =\theta \sqrt{P_{P-\text{Tx}}}x_{P}\left(h_{PS}+\sqrt{\alpha }h_{R_{k}S}h_{PR_{k}}\right)+\left(\sqrt{\alpha }h_{R_{k}S}n_{R_{k}}+n_{S}\right) \end{array}$$

where *h_PS_*, *h_PRk_*, *h_R_k_S_* which are modeled as a complex Gaussian random variable with zero mean denote the instantaneous channel gain of the channel from the primary user P to a secondary user S, from the primary user P to a best secondary relay R*_k_*, and from the best secondary relay R*_k_* to the secondary user S, respectively; *n_s_* and *n_Rk_* denote the additive Gaussian noise with zero-mean and unit variance.

Let *Y_S_*_,_*_AF_* = |*y_S_*_,_*_AF_*|^2^ be the output of the energy detector of the secondary user S. The expected signal power *γ_S_*_,_*_AF_* of *Y_S_*_,_*_AF_* is calculated as:

(21) $$\gamma_{S,AF}=E\left[Y_{S,AF}\right]=\theta \left(P_{S-Rx}+\alpha P_{R_{k}-Rx}{\left|h_{R_{k}S}\right|}^{2}\right)+\left(\alpha {\left|h_{R_{k}S}\right|}^{2}+1\right)$$

where *P_S_*_−_*_Rx_* and *P_Rk_*_−_*_Rx_* refer to the received signal power at secondary user S and secondary relay R*_k_* from the primary user P, respectively.

Let

$$z=\frac{{\left|h_{R_{k}S}\right|}^{2}/\psi_{R_{k}S}}{{\left|f_{R_{k}D}\right|}^{2}/\psi_{R_{k}D}}$$

as in Equation (12) and the *pdf* of *z* is given as in Equation (13). Define *ψ* = *ψ_R_k_S_*/*ψ_R_k_D_* Equation (21) is rewritten as

(22) $$\gamma_{S,AF}=(1+\theta P_{R_{k}-Rx})I_{t}\psi z+\theta P_{S-Rx}+1$$

Since *y_S_*_,_*_AF_* given *h_R_k_S_* and *f_R_k_D_* is complex Gaussian random variable, *Y_S_*_,_*_AF_* given *z* follows exponential distribution with parameter *1*/*γ_S_*_,_*_AF_*.

The detection probability *P_d,S,AF_* of S is given by

(23) $$\begin{array}{ll} P_{d,S,AF} & =\mathrm{Pr}\left(Y_{S,AF}>\lambda_{S,AF}|H_{1}\right)\overset{N}{\underset{k=1}{\text{∑}}}\mathrm{Pr}\left({\left|h_{R_{k}S}\right|}^{2}>\underset{h_{R_{i}S}\in R,i\neq k}{\max}{\left|h_{R_{i}S}\right|}^{2}\right)\\ & =\mathrm{Pr}\left(Y_{S,AF}>\lambda_{S,AF}|H_{1}\right)\overset{N}{\underset{k=1}{\text{∑}}}\Omega_{k}\\ & =\int_{0}^{\infty }\mathrm{Pr}\left(Y_{S,AF}>\lambda_{S,AF}|H_{1},z\right)f\left(z\right)dz\\ & =\int_{0}^{\infty }\int_{\lambda_{S,AF}}^{\infty }\frac{1}{{\left(1+z\right)}^{2}}\frac{1}{\gamma_{S,AF|H_{1}}}\exp\left(-\frac{x}{\gamma_{S,AF|H_{1}}}\right)\text{dxdz}\\ & =\int_{0}^{\infty }\frac{1}{{\left(1+z\right)}^{2}}\exp\left(\frac{-\lambda_{S,AF}}{(1+P_{R-Rx})I_{t}Gz+P_{S-Rx}+1}\right) \end{array}$$

where *λ_S_*_,_*_AF_* refers to the threshold of energy detector of the secondary user S.

The value of threshold *λ_S_*_,_*_AF_* in each sensing period is priory determined. We assume that all the secondary users have to maintain the same predefined false alarm probability before doing spectrum sensing, *i.e.*, *P_f_*_,_*_S_*_,_*_AF_* = *P_f_*_,_ *_Rk_* = *β* (the same value as in DF-CSS scheme) then the threshold *λ_S_*_,_*_AF_* is determined as follows

(24) $$\begin{array}{ll} P_{f,S,AF} & =\mathrm{Pr}\left(Y_{S,AF}>\lambda_{S,AF}|H_{0}\right)\overset{N}{\underset{k=1}{\text{∑}}}\Omega_{k}\\ & =\int_{0}^{\infty }\mathrm{Pr}\left(Y_{S,AF}>\lambda_{S,AF}|H_{0},z\right)f\left(z\right)dz\\ & =\int_{0}^{\infty }\int_{\lambda_{S,AF}}^{\infty }\frac{1}{{\left(1+z\right)}^{2}}\frac{1}{\gamma_{S,AF|H_{0}}}\exp\left(-\frac{x}{\gamma_{S,AF|H_{0}}}\right)\text{dxdz}.\\ & =\int_{0}^{\infty }\frac{1}{{\left(1+z\right)}^{2}}\exp\left(\frac{-\lambda_{S,AF}}{I_{t}\psi z+1}\right)dz \end{array}$$

### 3.4. Interference Probability

In this subsection, we take into account the case that the secondary relays have imperfect CSI of the interference link between the primary receiver D and the secondary relay R*_k_*. Let denote *f_R_k_D_*_,_*_im_* as the imperfect interference link between R*_k_* and D. Then, according to [14], *f_R_k_D_*_,_*_im_* is given as:

(25) $$f_{R_{k}D,im}=\rho f_{R_{k}D}+\tau \sqrt{1-\rho^{2}}$$

where *τ* is a circular symmetric complex Gaussian random variable with zero mean and *ψ_R_k_D_*/2 variance and *ρ* (0 < *ρ* < *1*) is the correlation coefficient between *f_R_k_D_* and *f_R_k_D_*_,_*_im_*.

Since transmission power at R*_k_* is rewritten as:

(26) $$P_{R_{k}-\text{Tx},im}=\frac{ɛI_{t}}{{\left|f_{R_{k}D,im}\right|}^{2}}$$

where *ε* (*ε* ≠ *0*) is control power coefficient used to adjust transmit power for analyzing the influence of secondary transmission on primary network.

consequently, by using the theorem of total probability, the interference probability *p_i_* is given as follows:

(27) $$\begin{array}{ll} P_{I} & =\mathrm{Pr}\left(P_{R_{k}-\text{Tx},im}{\left|f_{R'D}\right|}^{2}>I_{t}\right)\overset{N}{\underset{k=1}{\text{∑}}}\mathrm{Pr}\left({\left|h_{R_{k}S}\right|}^{2}>\underset{h_{R_{i}S}\in R,i\neq k}{\max}{\left|h_{R_{i}S}\right|}^{2}\right)\\ & =\mathrm{Pr}\left(P_{R_{k}-\text{Tx},im}{\left|f_{R'D}\right|}^{2}>I_{t}\right)\overset{N}{\underset{k=1}{\text{∑}}}\Omega_{k} \end{array}$$

Define:

(28) $$g\left(z\right)=\mathrm{Pr}\left(\frac{{\left|f_{R_{k}D}\right|}^{2}}{{\left|f_{R_{k}D,im}\right|}^{2}}>z\right)$$

The joint probability density function (*pdf*) of *f_R_k_D_* and *f_R_k_D_*_,_*_im_* is given as [14]:

(29) $$f_{{\left|f_{R_{k}D}\right|}^{2},{\left|f_{R_{k}D,im}\right|}^{2}}\;\left(x,y\right)=\frac{e^{-\frac{x+y}{\left(1-\rho^{2}\right)\psi_{R_{k}D}}}}{\left(1-\rho^{2}\right)\psi_{R_{k}D}^{2}}\;I_{0}\;\left(\frac{2\rho \sqrt{xy}}{\left(1-\rho^{2}\right)\psi_{R_{k}D}}\right)$$

where I_0_(.) is the zero th order modified Bessel function of the first kind.

We derive *g*(*z*) as follows:

(30) $$\begin{array}{ll} g(z) & =\int_{y=0}^{\infty }\int_{x=yz}^{\infty }\left(e^{-\frac{x+y}{\left(1-\rho^{2}\right)\psi_{R_{k}D}}}/\left(1-\rho^{2}\right)\psi_{R_{k}D}^{2}\right)\;I_{0}\;\left(\frac{2\rho \sqrt{xy}}{\left(1-\rho^{2}\right)\psi_{R_{k}D}}\right)\text{dxdy}\\ & \overset{\left(a\right)}{=}\int_{0}^{\infty }\frac{1}{\psi_{R_{k}D}}\exp\left(\frac{-y}{\psi_{R_{k}D}}\right)Q_{1}\left(\sqrt{\frac{2y}{\left(1-\rho^{2}\right)\psi_{R_{k}D}}},\sqrt{\frac{2yz}{\left(1-\rho^{2}\right)\psi_{R_{k}D}}}\right)dy\\ & \overset{\left(b\right)}{=}\frac{1}{2}\left(1+\frac{1-z}{\sqrt{{\left(1+z\right)}^{2}-4\rho^{2}z}}\right) \end{array}$$

where $Q(a,b)=\int_{b}^{\infty }x\exp\left(-\left(x^{2}+a^{2}\right)/2\right)I_{0}(ax)dx$ is the Marcum-Q function.

Herein, the equal sign (a) is obtained by making a change of variable, *i.e.*, $t=\sqrt{x}$, and with the help of Equation (9) in [15]. The equal sign (b) is obtained by using Equation (55) from [15]. From Equation (27), (28) and (30), interference probability *P_I_* is derived as:

(31) $$P_{I}=g\left(\frac{1}{ɛ}\right)$$

Notice that *ε* (*ε* ≠ *0*) is control power coefficient used to adjust transmit power for analyzing the influence of best relay's transmission on the primary receiver D.

## 4. Performance Evaluation

In this section, we present the performance evaluation of our proposed schemes with the results of simulation and analysis under various different scenarios. In both DF-CSS and AF-CSS, we assume that S is the secondary user that has the weaker ability to sense the primary spectrum than the secondary relay R*_k_*, *i.e.*, *P_Rk_*_−_*_Rx_* > *P_S_*_−_*_Rx_*, where *P_Rk_*_−_*_Rx_* and *P_S_*_−_*_Rx_* are the received power from the primary user P at the secondary user R*_k_* and S, respectively. The average channel gains *ψ_R_k_S_* and *ψ_R_k_D_* are modeled as a function of *P_Rk_*_−_*_Rx_* and *P_S-Rx_*, and of *P_Rk_*_−_*_Rx_* and *P_D-Rx_*, respectively. We assume that the primary user P, the secondary relays and the secondary user are nearly collinear. This is practical since the secondary relays are assumed to be closely located in a small area and the distance between the primary user P, the secondary user S and the area that the secondary relay R*_k_* are located are far enough away. For simplicity, the received signal powers at user *i* (*i*^∈^{S, R*_k_*}) from the primary user P are modeled as [7] *P_i_* = (*d_i_*)^–3^, where *d_i_* is the distance between user *i* and P. Thus, *ψ_R_k_S_* and *ψ_R_k_D_* are obtained as:

(32) $$\begin{array}{cc} \psi_{R_{k}S}=\frac{1}{{\left|d_{R_{k}}-d_{S}\right|}^{3}}, & \psi_{R_{k}D}=\frac{1}{{\left|d_{R_{k}}-d_{D}\right|}^{3}} \end{array}$$

Where *d_Rk_* is the distance between secondary user R*_k_* and P, and *d_S_* is the distance between secondary user S and P, and *d_D_* is the distance between primary receiver D and primary user P.

Notice that, in this paper, the average SNRs of the primary signal at R*_k_*'s are assumed to be identical. Without loss of generality, the pre-assigned false alarm probability *β* of all secondary users is always set as *β* = 0.1 and the received power at D and S from P is always *P_D-Rx_* = 3 dB and *P_S-Rx_* = 0 dB, respectively.

In a cognitive radio network, when the primary users are detected, the secondary users have to leave that channel immediately. This makes the detection probability important to the primary users since the detection probability determines the primary user's degree protection from secondary users. Hence, the detection probability should be maintained at a high level. In this section, we will discuss the detection probability of the secondary user S as the sensing performance and on the main role of interference threshold *I_t_* in the proposed schemes.

**Figure 2.**
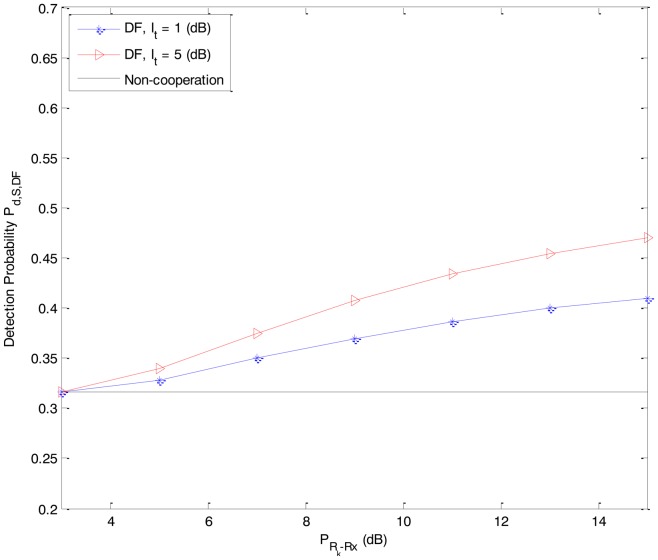
Detection probability *P_d,S,DF_* as a function of the received power *P_Rk_*_−_*_Rx_* from the primary user P at the secondary user R*_k_* in the DF-CSS scheme (P*_D-Rx_* = 3 dB, P*_S-Rx_* = 0 dB).

**Figure 3.**
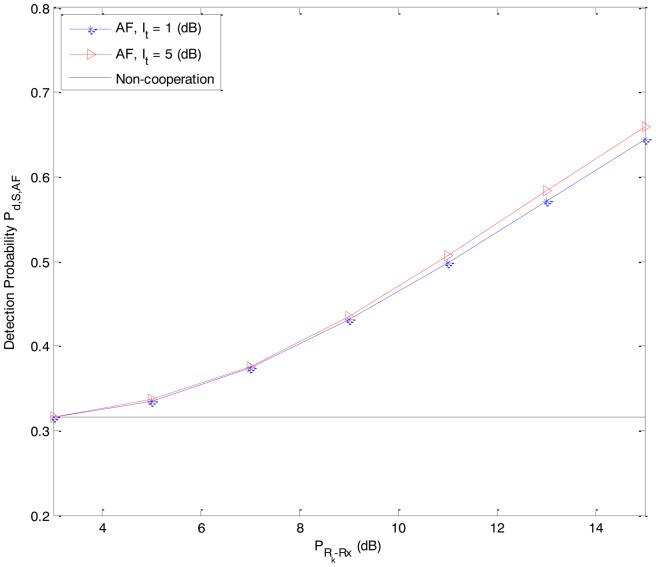
Detection probability *P_d,S,AF_* as a function of the received power *P_Rk_*_−_*_Rx_* from the primary user P at the secondary user R*_k_* in the AF-CSS scheme (P*_D-Rx_* = 3 dB, P*_S-Rx_* = 0 dB).

In Figures 2 and 3, we present the detection probability *P_d,S,DF_* and *P_d,S,AF_* as functions of the received power *P_Rk_*_−_*_Rx_* from the primary user P at the secondary user R*_k_* of the DF-CSS scheme and AF-CSS scheme, respectively. As we can see, according to the increasing value of *P_Rk_*_−_*_Rx_*, the detection probability of S is better since R*_k_* gets higher received power. Especially, at a given value of *P_Rk_*_−_*_Rx_* the detection probability increases when the value of *I_t_* is increased.

**Figure 4.**
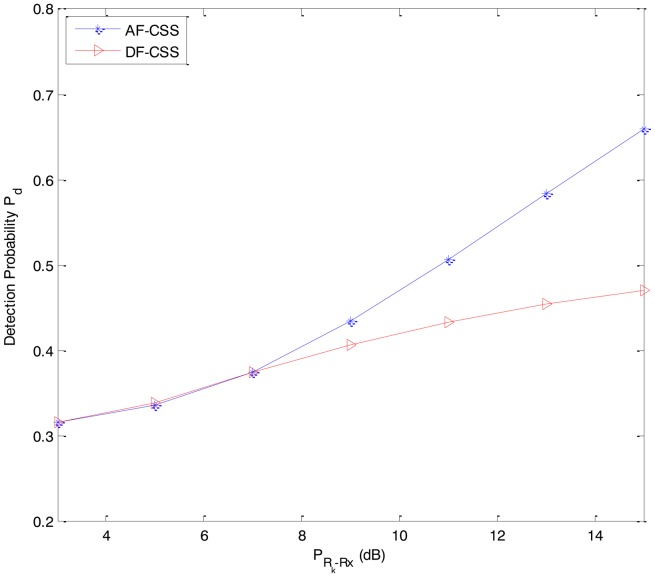
Detection probability *P_d_* between the DF-CSS and AF-CSS schemes as a function of the received power *P_Rk_*_−_*_Rx_* from the primary user P at the secondary user R*_k_* (P*_D-Rx_* = 3 dB, P*_S-Rx_* = 0 dB, *I_t_* = 5 dB).

In Figure 4, we compare the sensing performance (detection probability) between DF-CSS and AF-CSS under the same parameter such that *I_t_* = 5 dB. As we can see in Figure 4, the sensing performance (detection probability) of the AF-CSS scheme is superior to the DF-CSS scheme at high value of *P_Rk_*_−_*_Rx_* (*i.e.*, *P_Rk_*_−_*_Rx_* is larger than 7 dB). However, at low value of *P_Rk_*_−_*_Rx_* (*i.e.*, *P_Rk_*_−_*_Rx_* is from 3 dB to 7 dB), the sensing performance (*i.e.*, detection probability) is almost the same for the DF-CSS and AF-CSS schemes.

**Figure 5.**
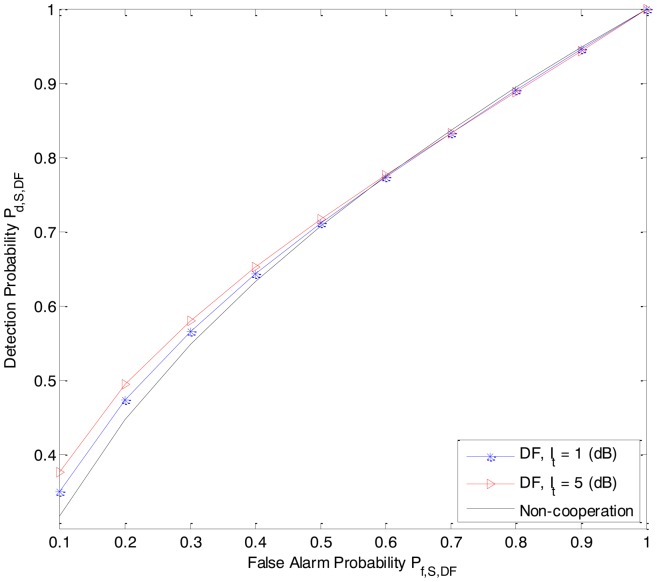
The ROC (*P_d,S,DF_* vs. *P_f,S,DF_*) of the secondary user S in the DF-CSS scheme (P*_D-Rx_* = 3 dB, P*_S-Rx_* = 0 dB, *P_Rk_*_−_*_Rx_* = 7 dB).

**Figure 6.**
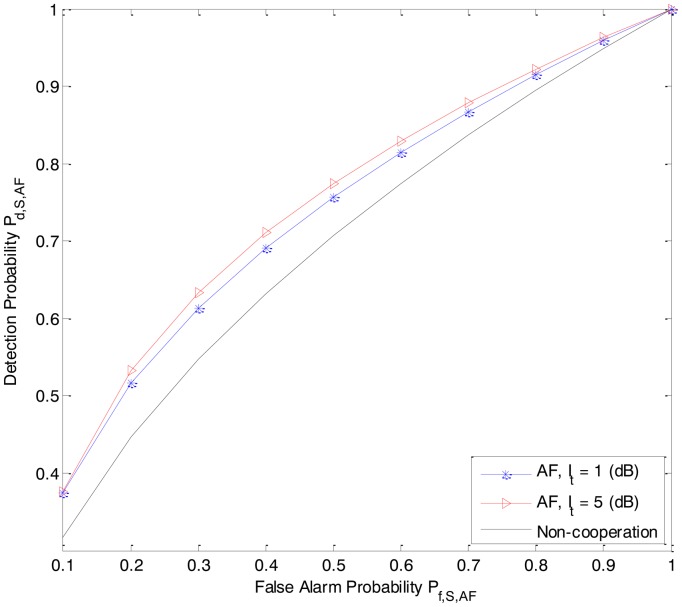
The ROC (*P_d,S,AF_* vs. *P_f_*_,_*_S_*_,_*_AF_*) of the secondary user S in the AF-CSS scheme (P*_D-Rx_* = 3 dB, P*_S-Rx_* = 0 dB, *P_Rk_*_−_*_Rx_* = 7 dB).

In Figure 5 and Figure 6, we quay the sensing performance by depicting the receiver operating characteristic (ROC) (detection probability *P_d_*_,_ (*i.e.*, *P_d,S,DF_* and *P_d,S,AF_*) *versus* false alarm probability *P_f_* (*i.e.*, *P_f,S,DF_* and *P_f_*_,_*_S_*_,_*_AF_*)) for the DF-ASS and AF-CSS schemes, respectively, in which, *P_Rk_*_−_*_Rx_* = 7 dB, interference threshold *I_t_* is 1 dB and 5 dB, respectively. In the context of cognitive radio, the value of P*_f_* should be maintained at a low level since the false alarm probability determines the percentage of the white spaces that are misclassified as occupied. Hence, we just consider the sensing performance of the secondary user S at a low level of false alarm probability of S. As we can see, for a given value in low false alarm probability region, the detection probabilities in both the DF-CSS and AF-CSS schemes increase when value of *I_t_* is increased.

From Figure 2^3^, 4, 5 to Figure 6, we can see that in both the DF-CSS and AF-CSS schemes, with the same received power from the primary user at secondary user R*_k_* and S, the detection probability is increased according to the increase of the interference threshold *I_t_*. It means that when the relay is allowed to send the data to the secondary user S with higher transmission power, the final sensing result at the secondary user S is more reliable. Moreover, we can see that the sensing performance of both the schemes is improved if the received power from the primary user at the secondary relay R*_k_* is increased. Obviously, it may be explained that the sensing reliability is higher when the relay receives the stronger signal from primary user.

**Figure 7.**
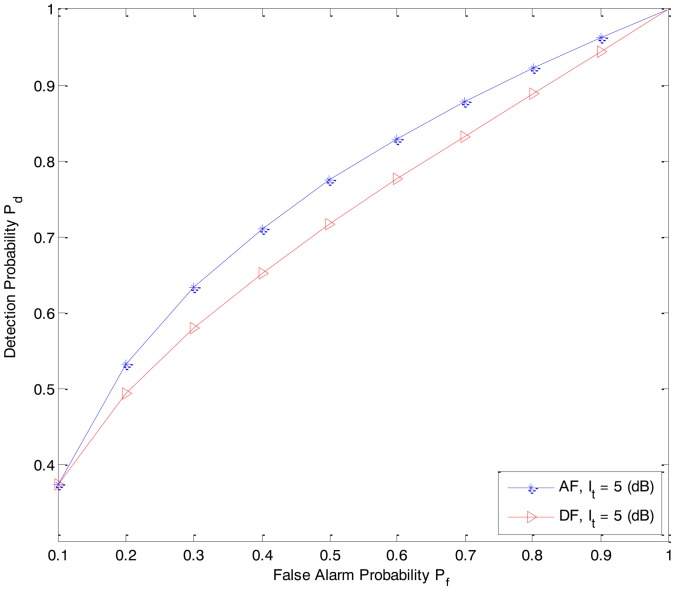
Comparison of the ROC (*P_d_* vs. *P_f_*) of the secondary user S between DF-CSS and AF-CSS (P*_D-Rx_* = 3 dB, P*_S-Rx_* = 0 dB, *P_Rk_*_−_*_Rx_* = 7 dB, *I_t_* = 5 dB).

In Figure 7, we compare the sensing performance of the DF-CSS and AF-CSS schemes. At the point of *P_f_* = 0.1, both the schemes have the same sensing performance. However, when *P_f_* increases, the AF-CSS scheme is superior than the DF-CSS scheme.

**Figure 8.**
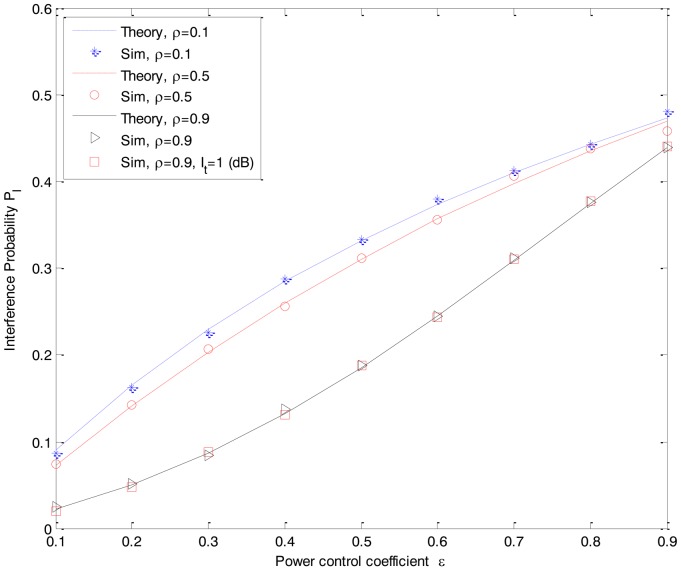
Interference probability *P_I_* between secondary and primary networks as a function of the transmit power control coefficient *ε* of the secondary user R*_k_*. (P*_D-Rx_* = 3 dB, P*_S-Rx_* = 0 dB, *P_Rk_*_−_*_Rx_* = 7 dB, *I_t_* = 5 dB).

Figure 8 shows the interference probability *P_I_* of the both the DF-CSS and AF-CSS schemes as a function of the transmission power control coefficient *ε* of the secondary user R*_k_*. Herein, without loss of generality, we choose the standard *I_t_* = 5 dB and *P_Rk_*_−_*_Rx_* = 3 dB. As we can see in Figure 8, the interference of the secondary transmission to the primary network becomes higher when we increase the transmission power control coefficient *ε* which makes the transmission power of R*_k_* increase. Notice that the transmission power of the secondary relay R*_k_* is determined based on the interference threshold *I_t_*. However, when the condition of the interference link between secondary R*_k_* and primary D is better (*i.e.*, *ρ* is higher, where *ρ* is the correlation coefficient between *f_R_k_D_* and *f_R_k_D_*_,_*_im_*), the interference probability *P_I_* is decreased. The reason is that with more exact information about the primary receiver, the interference caused by a secondary network on the primary network is reduced. In addition, the interference threshold *I_t_* itself does not impact on the change of interference probability *P*_I_. As we can see in Figure 8, the scenario with *ρ* = 0.9 and *I_t_* = 1 dB gives the same simulation result as in the scenario with *ρ* = 0.9 and *I_t_* = 5 dB in both the DF-CSS and AF-CSS schemes.

## 5. Conclusions

In this paper, we propose two soft decision cooperative spectrum sensing schemes using the combination of an underlay cognitive radio approach and a best relay selection scheme. Through theoretical analysis and simulation results, we can reach the following conclusions: detection probability depends on the value of interference threshold *I_t_* at a primary user. In both the DF-CSS and AF-CSS schemes, with the same received power from the primary user at a secondary user R*_k_* and S, we can see that the sensing performances are increased according to the increase of the interference threshold *I_t_*. Moreover, we can see that the sensing performance of the both schemes is improved if the received power from the primary user at the secondary relay R*_k_* is increased. The interference caused by secondary users on the primary operation is characterized by three main parameters, which are interference probability *P_I_*, the correlation coefficient *ρ* between *f_R_k_D_* and *f_R_k_D_*_,_*_im_*, and the control power coefficient *ε*. For a given *ε*, the interference probability *P_I_* is increased as the correlation coefficient *ρ* decreases. It means that the worse CSI the secondary users estimate, the more interference the secondary transmission causes on the primary operation. On the other hand, for the same received power from the primary user, interference caused by data transmission of R*_k_* on a primary user is increased according to the increased transmission power of the secondary relay R*_k_* which is adjusted by using the control power coefficient *ε* in both AF-CSS and DF-CSS schemes. Therefore, we need a tradeoff between the sensing reliability and the interference between primary and secondary networks because we adopt the underlay approach in spectrum sensing in cognitive radio networks.

## Appendix 1

### Derivation of Equation (17)

$$\begin{array}{ll} P_{d,S,DF} & =\mathrm{Pr}\left(Y_{S,DF}>\lambda_{S,DF}|H_{1}\right)\overset{N}{\underset{k=1}{\text{∑}}}\Omega_{k}\\ & =\mathrm{Pr}\left(Y_{S,DF}(\theta_{R_{k}}=1)>\lambda_{S,DF}|\theta_{R_{k}}=1,H_{1}\right)\mathrm{Pr}\left(\theta_{R_{k}}=1|H_{1}\right)\\ & +\mathrm{Pr}\left(Y_{S,DF}(\theta_{R_{k}}=0)>\lambda_{S,DF}|\theta_{R_{k}}=0,H_{1}\right)\mathrm{Pr}\left(\theta_{R_{k}}=0|H_{1}\right)\\ & =\mathrm{Pr}\left(Y_{S,DF}(\theta_{R_{k}}=1)>\lambda_{S,DF}|H_{1}\right)\mathrm{Pr}\left(Y_{R_{k}}>\lambda_{R_{k}}|H_{1}\right)\\ & +\mathrm{Pr}\left(Y_{S,DF}(\theta_{R_{k}}=0)>\lambda_{S,DF}|H_{1}\right)\left(1-\mathrm{Pr}\left(Y_{R_{k}}>\lambda_{R_{k}}|H_{1}\right)\right)\\ & =P_{f,R_{k}}\int_{0}^{\infty }\mathrm{Pr}\left(Y_{S,DF}(\theta_{R_{k}}=1)>\lambda_{S,DF}|H_{1},z\right)f(z)dz\\ & +\left(1-P_{f,R_{k}}\right)\int_{0}^{\infty }\mathrm{Pr}\left(Y_{S,DF}(\theta_{R_{k}}=0)>\lambda_{S,DF}|H_{1},z\right)f(z)dz\\ & =\exp\left(-\frac{\lambda_{R_{k}}}{P_{R_{k}-Rx}+1}\right)\int_{0}^{\infty }\int_{\lambda_{S,DF}}^{\infty }\frac{1}{{\left(1+z\right)}^{2}}\frac{1}{\gamma_{S,DF|H_{1},\theta_{R_{k}}=1}}\exp\left(\frac{-x}{\gamma_{S,DF|H_{1},\theta_{R_{k}}=1}}\right)\text{dxdz}\\ & +\left(1-\exp\left(-\frac{\lambda_{R_{k}}}{P_{R_{k}-Rx}+1}\right)\right)\int_{0}^{\infty }\int_{\lambda_{S,DF}}^{\infty }\frac{1}{{\left(1+z\right)}^{2}}\frac{1}{\gamma_{S,DF|H_{1},\theta_{R_{k}}=0}}\exp\left(\frac{-x}{\gamma_{S,DF|H_{1},\theta_{R_{k}}=0}}\right)\text{dxdz}\\ & =P_{d,R_{k}}\int_{0}^{\infty }\int_{\lambda_{S,DF}}^{\infty }\frac{1}{{\left(1+z\right)}^{2}}\frac{1}{\gamma_{S,DF|H_{1},\theta_{R_{k}}=1}}\exp\left(\frac{-x}{\gamma_{S,DF|H_{1},\theta_{R_{k}}=1}}\right)\text{dxdz}\\ & +\left(1-P_{d,R_{k}}\right)\int_{0}^{\infty }\int_{\lambda_{S,DF}}^{\infty }\frac{1}{{\left(1+z\right)}^{2}}\frac{1}{\gamma_{S,DF|H_{1},\theta_{R_{k}}=0}}\exp\left(\frac{-x}{\gamma_{S,DF|H_{1},\theta_{R_{k}}=0}}\right)\text{dxdz}\\ & =\beta^{\frac{1}{P_{R_{k}-Rx}+1}}\int_{0}^{\infty }\frac{1}{{\left(1+z\right)}^{2}}\exp\left(\frac{-\lambda_{S,DF}}{I_{t}\psi z+P_{S-Rx}+1}\right)dz\\ & +\left(1-\beta^{\frac{1}{P_{R_{k}-Rx}+1}}\right)\exp\left(\frac{-\lambda_{S,DF}}{P_{S-Rx}+1}\right). \end{array}$$

## Appendix 2

### Derivation of Equation (18)

$$\begin{array}{ll} P_{f,S,DF} & =\mathrm{Pr}\left(Y_{S,DF}>\lambda_{S,DF}|H_{0}\right)\overset{N}{\underset{k=1}{\text{∑}}}\Omega_{k}\\ & =\mathrm{Pr}\left(Y_{S,DF}(\theta_{R_{k}}=1)>\lambda_{S,DF}|\theta_{R_{k}}=1,H_{0}\right)\mathrm{Pr}\left(\theta_{R_{k}}=1|H_{0}\right)\\ & +\mathrm{Pr}\left(Y_{S,DF}(\theta_{R_{k}}=0)>\lambda_{S,DF}|\theta_{R_{k}}=0,H_{0}\right)\mathrm{Pr}\left(\theta_{R_{k}}=0|H_{0}\right)\\ & =\mathrm{Pr}\left(Y_{S,DF}(\theta_{R_{k}}=1)>\lambda_{S,DF}|H_{0}\right)\mathrm{Pr}\left(Y_{R_{k}}>\lambda_{R_{k}}|H_{0}\right)\\ & +\mathrm{Pr}\left(Y_{S,DF}(\theta_{R_{k}}=0)>\lambda_{S,DF}|H_{0}\right)\left(1-\mathrm{Pr}\left(Y_{R_{k}}>\lambda_{R_{k}}|H_{0}\right)\right)\\ & =P_{f,R_{k}}\int_{0}^{\infty }\mathrm{Pr}\left(Y_{S,DF}(\theta_{R_{k}}=1)>\lambda_{S,DF}|H_{0},z\right)f(z)dz\\ & +\left(1-P_{f,R_{k}}\right)\int_{0}^{\infty }\mathrm{Pr}\left(Y_{S,DF}(\theta_{R_{k}}=0)>\lambda_{S,DF}|H_{0},z\right)f(z)dz\\ & =P_{f,R_{k}}\int_{0}^{\infty }\int_{\lambda_{S,AF}}^{\infty }\frac{1}{{\left(1+z\right)}^{2}}\frac{1}{\gamma_{S,DF|H_{0},\theta_{R_{k}}=1}}\exp\left(-\frac{x}{\gamma_{S,DF|H_{0},\theta_{R_{k}}=1}}\right)\text{dxdz}\\ & +\left(1-P_{f,R_{k}}\right)\int_{0}^{\infty }\int_{\lambda_{S,AF}}^{\infty }\frac{1}{{\left(1+z\right)}^{2}}\frac{1}{\gamma_{S,DF|H_{0},\theta_{R_{k}}=0}}\exp\left(-\frac{x}{\gamma_{S,DF|H_{0},\theta_{R_{k}}=0}}\right)\text{dxdz}\\ & =\left(1-\beta \right)\exp\left(-\lambda_{S,DF}\right)+\beta \int_{0}^{\infty }\frac{1}{{\left(1+z\right)}^{2}}\exp\left(\frac{-\lambda_{S,DF}}{I_{t}\psi z+1}\right)dz. \end{array}$$
